# Microbial Utilization of Next-Generation Feedstocks for the Biomanufacturing of Value-Added Chemicals and Food Ingredients

**DOI:** 10.3389/fbioe.2022.874612

**Published:** 2022-04-11

**Authors:** Congqiang Zhang, Christoph Ottenheim, Melanie Weingarten, LiangHui Ji

**Affiliations:** ^1^ Singapore Institute of Food and Biotechnology Innovation (SIFBI), Agency for Science, Technology and Research (A*STAR), Singapore, Singapore; ^2^ Temasek Life Sciences Laboratory, National University of Singapore, Singapore, Singapore

**Keywords:** C1 feedstocks, C2 feedstocks, metabolic engineering, synthetic biology, CO_2_ utilization

## Abstract

Global shift to sustainability has driven the exploration of alternative feedstocks beyond sugars for biomanufacturing. Recently, C1 (CO_2_, CO, methane, formate and methanol) and C2 (acetate and ethanol) substrates are drawing great attention due to their natural abundance and low production cost. The advances in metabolic engineering, synthetic biology and industrial process design have greatly enhanced the efficiency that microbes use these next-generation feedstocks. The metabolic pathways to use C1 and C2 feedstocks have been introduced or enhanced into industrial workhorses, such as *Escherichia coli* and yeasts, by genetic rewiring and laboratory evolution strategies. Furthermore, microbes are engineered to convert these low-cost feedstocks to various high-value products, ranging from food ingredients to chemicals. This review highlights the recent development in metabolic engineering, the challenges in strain engineering and bioprocess design, and the perspectives of microbial utilization of C1 and C2 feedstocks for the biomanufacturing of value-added products.

## Introduction

The COP26 UN climate change summit (https://ukcop26.org/) has set the goal to reach net zero carbon emission by the middle of this century. The global shift from fossil fuels to more sustainable and green resources and technology has offered a great opportunity for biomanufacturing, particularly microbial fermentation. Although current industrial fermentation processes heavily rely on carbohydrate substrates like glucose and sucrose, microbes have the capability or potentials to use C1 substrates (carbon dioxide, carbon monoxide, methane, methanol and formate) (Schrader et al., 2009; Jiang et al., 2021) and C2 substrates (mainly ethanol and acetate) (Kiefer et al., 2020). C1 and C2 substrates are inexpensive, either naturally abundant, easy to produce or available as industrial wastes and by-products. More importantly, their utilization does not compete with food sources; supports a sustainable economy; and reduces carbon emission to the environment. Hence, it aligns highly with the net zero target set forth by the COP26 summit.

The main challenge in using C1 and C2 feedstocks, collectively referred as next-generation feedstocks (NGFs), lies in the inefficiency in assimilating into biomass and bioproducts by natural microbes. To overcome the technological challenges, researchers in metabolic engineering and synthetic biology have intelligently engineered and evolved microorganisms in laboratories to make best use of NGFs. For C1 feedstocks, current efforts are mainly spared on how to assimilate them faster and better into biomass and central metabolism of both naturally occurring microbes (e.g., chemoorganoautotrophs, methylotrophs) or synthetic model microbes, e.g., *Escherichia coli* (Antonovsky et al., 2016; Yu and Liao, 2018; Gleizer et al., 2019; Chen F. Y.-H. et al., 2020; Chou et al., 2021) and yeasts (Espinosa et al., 2020; Gassler et al., 2020). In contrast, industrial workhorse microbes can readily use C2 feedstocks without sophisticated genetic engineering or prolonged adaptive laboratory evolution (ALE). As such, C2 feedstocks have been directly used to produce value-added products, such as lipids (Park et al., 2019), isoprenoids (Yang and Nie, 2016), poly(3-hydroxybutyrate) (PHB) (Liang et al., 2021). For both C1 and C2 feedstocks, once they are assimilated into the central metabolic pathways (e.g., glycolysis, the tricarboxylic acid cycle) by microbes, the central metabolites such as acetyl-CoA or pyruvate can be readily redirected to synthesize value-added products, ranging from nutrients in food and feed, e.g., alternative proteins (Sillman et al., 2019), lipids (Bar-On and Milo, 2019), starch (Cai et al., 2021) and nutraceuticals (Zhang and Too, 2020), to chemicals, e.g., personal-care chemicals (Chen X. et al., 2020), pharmaceuticals (Shukal et al., 2019), agrochemicals (Kildegaard et al., 2021) and biofuels (Godar et al., 2021).

Due to the strong incentives in addressing global food shortage, climate change and sustainability issues and the tremendous efforts from academy and industry, the field in using NGF has advanced rapidly in the past decade. Here, we aim to present a brief summary on the very recent achievements focusing on the past 2–3 years in metabolic engineering and synthetic biology that use various NGFs for the production of biomass and/or value-added products. Unlike existing reviews focusing on either C1 (Cotton et al., 2020; Jiang et al., 2021) or C2 (Ma et al., 2022) feedstocks, we aim to compare the advantages and disadvantages of different C1 and C2 feedstocks and discuss their biotechnological potentials. We separately discuss natural metabolic pathways and synthetic routes of NGF assimilation using metabolic engineering and ALE strategies. We also discuss the topic from a different perspective in a holistic bioprocess evaluation that balances both biological and commercialization challenges, such as balancing carbon yields and productivity; counterweight of feedstock cost, pretreatment, usage efficiency and product diversification. We compare several strategies and argue that integrated bioprocesses may reach industrial applications earlier while single (one-bioreactor-and-one-strain) systems will require longer or more intriguing research. Finally, we highlight challenges and future perspectives in preparing microbial cell factories for industrial biomanufacturing.

## Overview of Various Next-Generation Feedstockss and Their Potentials in Industrial Biotechnology

Here, we refer C1 NGFs as C1 gas (CO_2_, CO and CH_4_), methanol and formic acid. As a major contributor to global warming, carbon dioxide (CO_2_) makes up 0.041% of Earth’s atmosphere and its concentration is still increasing due to human activities from burning fossil fuels for electricity, heat, and transportation (Luderer et al., 2018) and carbon emissions from the melting of Arctic permafrost that stores 1.7 trillion metric tons of carbon (Miner et al., 2022). As CO_2_ can be obtained from atmosphere or from industrial waste streams (e.g., flue gas), the cost of CO_2_ can be very low and even negative after carbon credits are factored in (Table 1). The current carbon emission tax in G20 economies is between $3 and $60 per ton (Routers report on 25 October 2021). Carbon capture cost can vary markedly by CO_2_ source, from a range of $15–25/ton CO_2_ for industrial processes producing “pure” or highly concentrated CO_2_ streams (such as natural gas processing or ethanol production) to $40–120/ton CO_2_ for processes with “dilute” gas streams, such as cement production and power generation (https://www.iea.org/commentaries/is-carbon-capture-too-expensive). This suggests that CO_2_ could be obtained at zero cost in some countries. Carbon monoxide (CO) is scarce in the atmosphere but can be efficiently produced from CO_2_ with the emerging CO_2_ electrolysis technology (Liew et al., 2016). Also, CO is available as a waste gas in industrial processes from the partial oxidation of carbon-containing compounds and from gasification of waste stream (syngas, together with CO_2_ and H_2_) (Claassens et al., 2016). CO can also be produced by co-electrolysis of CO_2_ and H_2_O (Herranz et al., 2020). The primary concern in using CO is its high toxicity and difficulty to trace as it is colorless, odourless, and tasteless. Methane (CH_4_) is abundant in nature, especially in the form of natural and shale gas. In addition, methane is produced by human activities in larger amount than natural production, such as landfills, agricultural activities (e.g., animal livestock emissions and paddy rice cultivation), coal mining, wastewater treatment (Karakurt et al., 2012) (Table 1). In fact, anthropogenic methane contributes to at least 25% of today’s global warming, according to the Environmental Defense Fund estimation. This is because methane is a more powerful greenhouse gas, with approximately 20 times the impact of carbon dioxide (Strong et al., 2015). Hence, developing biotechnological use of methane and like CO_2_ has double meanings in revalorization (generating higher values than its primary use in generating electricity or heat) and reducing greenhouse emission.

**TABLE 1:** Advantages and disadvantages of various next-generation feedstocks (NGSs) and sugars.

Feedstocks	Chemical formula	Water solubility [g/L]^a^	Price ($/ton) ^b^	Sources	Advantages and uniqueness	Disadvantages
Carbon dioxide	CO_2_	1.69	0–80^c^	Earth’s atmosphere; human activities, e.g., burning fossil fuels for electricity, heat, and transportation; Arctic permafrost thawing	⁃Naturally abundant and free and even get carbon credit by reducing CO_2_ release	⁃Most oxidized, and zero reducing power (Figure 1B), requiring large amount of reducing power supply and hydrogen source from water, methanol (Lebloas et al., 1996) or H_2_
					⁃Non-toxic and non-flammable	⁃Very low solubility in water that limits mass transfer and microbial productivity
					⁃Tremendous efforts. and technological breakthrough from academy and industry	⁃Difficulty in storage and transportation
					⁃Breakthrough in CO_2_-fixing biotechnology in synthetic microbes	
					⁃Well explored in gas fermentation using anaerobic acetogens (Liew et al., 2016)	
Carbon monoxide	CO	0.028	27–298^d^	Industrial waste and electrosynthesis of CO_2_	⁃Can provide reducing equivalent in the Wood-Ljungdahl Pathway for CO_2_ assimilation	⁃Lower reducing power and requiring additional reducing power supply
					⁃A diverse group of bacteria and archaea, referred to as carboxydotrophs, can use CO as a primary carbon and energy source	⁃Very low solubility in water that limits mass transfer and microbial productivity
					⁃Well explored in gas fermentation using anaerobic acetogens (Liew et al., 2016)	⁃Difficulty in storage and transportation
						⁃Toxic, flammable and explosive
Methane	CH_4_	0.023	200–320^e^	Natural and shale gas; syngas and human activities e.g., landfills, agricultural activities, coal mining, wastewater treatment	⁃Naturally abundant and low price	⁃Very low solubility in water that limits mass transfer and microbial productivity
					⁃Highest degree of reduction, energy intensive	⁃Difficulty in storage and transportation
					⁃Naturally used by methanotrophs	⁃Flammable and explosive
					⁃Can be used as sole feedstock to supply both carbon and energy	⁃Challenges in heterologous expression of methane monooxygenases (MMOs) (Jiang et al., 2021)
						⁃Challenges in engineering methanotrophs
Methanol	CH_4_OH	Miscible	150–300^f^	Synthesis from natural gas, syngas and hydrogenation of CO_2_	⁃A bulk chemical and relatively cheap	⁃Formation of the very toxic intermediate formaldehyde so that methanol concentration must be kept low (∼5 g/L)
					⁃Higher degree of reduction and electron rich	⁃Low productivity by wildtype methylotrophs and engineered biotechnological microbes while used as sole feedstock. Currently, the shortest reported doubling time is 8.5 h in an evolved *E. coli* (Chen et al., 2020b)
					⁃Completely water miscible and higher mass transfer and supports higher microbial productivities	⁃Flammable
					⁃Easy transportation and storage	⁃More research efforts are required for faster assimilation of methanol in microbes
					⁃Can be used as sole feedstock to supply both carbon and energy	⁃High fermentation cost required to neutralize the heat generated by methanol oxidation
					⁃Higher energetic efficiency as compared to H_2_/CO_2_ or CO when used by acetogens (Claassens et al., 2019)	⁃High oxygen demand
Formic acid	HCOOH	972	450–500^g^	Electrochemical, photoreduction of CO_2_, or hydrogenation of CO_2_	⁃High solubility in water and other polar solvents, higher mass transfer and supports higher microbial productivities	⁃Relatively higher price than methanol
					⁃Inflammable and higher degree of reduction than CO_2_ and CO.	⁃Formation of the toxic intermediate formaldehyde when assimilated by microbes
					⁃Easy transportation and storage	⁃Less studied as compared to methanol as microbial feedstock
					⁃Higher energetic efficiency as compared to H_2_/CO_2_ or CO when used by acetogens (Claassens et al., 2019)	⁃More oxidized than methanol and thus less reducing power
						⁃Low productivity while used as main feedstock, doubling time is 65.9 h in a highly engineered *E. coli* (Bang et al., 2020)
						⁃More research efforts are required for faster assimilation of formate in microbes
						⁃Alkali is required to neutralize the acidity as in aerobic fermentation, weak acids are a powerful respiratory uncoupler so have to be used under strict carbon limiting conditions and relatively high pH to limit the amount of free formic acid
Ethanol	C_2_H_5_OH	Miscible	250–350^h^	fermentation from starch based raw materials and lignocellulose	⁃A bulk chemical and relatively cheap	⁃Relatively more expensive than methanol
					⁃Great advance in bio-ethanol technology	⁃The technology of cellulosic ethanol should be further improved
					⁃High solubility in water, higher mass transfer and supports higher microbial productivities	⁃Further boosting the productivity of microbes growing on ethanol
					⁃Easy assimilation by industrial workhorse microorganisms	⁃Metabolic engineering efforts are required to further boost the conversion yield of ethanol to high-value bioproducts
					⁃Can be fed into bioreactor in pure form	⁃Flammable
					⁃Produce acetyl-CoA, a key precursor for several value-added bioproducts (e.g., lipids, terpenoids, polyketides, PHB) (Kiefer et al., 2020)	⁃High fermentation cost required to neutralize the heat generated by ethanol oxidation
						⁃High oxygen demand
Acetic acid	CH_3_COOH	1,233	300–450^i^	Methanol carbonylation, sugar fermentation, depolymerization of lignocellulose and acetogen fermentation from C1 gas	⁃Natural product found in animal metabolism and food	⁃More expensive than methanol and ethanol
					⁃Lower toxicity than C1 chemicals	⁃Technology of bio-acetate should be further improved
					⁃A bulk chemical and with increasing global market, and bio-acetic acid market is growing rapidly	⁃Further boosting the growth rate and productivity of microbes growing on acetate
					⁃High solubility in water and other polar solvents, higher mass transfer and supports higher microbial productivities	⁃Metabolic engineering efforts are required to further boost the conversion yield of acetate to high-value bioproducts
					⁃Easy assimilation by industrial workhorse microorganisms	⁃As a respiratory uncoupler, alkali is required to neutralize the acidity of acetate and minimise substrate toxicity
					⁃Can be fed into bioreactor in pure form	⁃Central metabolism topology needs fine tuning to improve growth efficiency
					⁃Acetate is the direct precursor for acetyl-CoA that is used for the biosynthesis of numerous products (e.g., lipids, terpenoids, polyketides, PHB) (Kiefer et al., 2020)	
Glucose	C_6_H_12_O_6_	909	300–400	Hydrolysis of starch from corn, potato, wheat, and cassava	⁃Well established metabolic systems in microorganisms	⁃Competing with food source
					⁃Extensive knowledge on metabolism	⁃Releasing high-amount CO_2_ during fermentation process as compared to NGFs
					⁃High efficiency for microbial fermentation	
					⁃Non-toxic and non-flammable and easier to transport as solids	

a Solubility of next generation feedstocks in water at 1 atm pressure and 293 K (Kaye and Laby, 1986).

b The current prices for methane, methanol, ethanol and acetate are considerably higher than 1–2 years ago due to the global supply chain disruption, which is caused by COVID pandemic and political tensions, here we use the median price of pre-COVID period.

c The price is adjusted by factoring in carbon credit, https://www.reuters.com/business/cop/carbon-needs-cost-least-100tonne-now-reach-net-zero-by-2050-2021-10-25/

d Price is based on syngas, refer to Table 1, Biotechnol Biofuels. 2017; 10: 150.

e Gas lower heating value (lhv) is assumed for ship fuel based on 1 $/mmBTU (lhv)= 46.76 $/ton, https://www.dnv.com/maritime/insights/topics/lng-as-marine-fuel/current-price-development-oil-and-gas.html

f https://www.methanol.org/methanol-price-supply-demand/

g https://www.echemi.com/produce/pr2106011005-formic-acid-99-powder-saa6598-saa.html

h https://tradingeconomics.com/commodity/ethanol

i https://www.echemi.com/produce/pr2104271858-glacial-acetic-acid.html

The gaseous C1 feedstock can be derived directly from organic material by gasification in a controlled conversion process at high temperatures (Zhang Y. et al., 2020). A similar gas mixture consisting of CO and H_2_ can be produced by steam reforming from CH_4_ (Chen L. et al., 2020). In both cases the resulting gas is termed syngas and can be utilized as an NGF. Also, incineration of waste streams can generate large amounts of syngas, which is less exploited currently.

**FIGURE 1:**
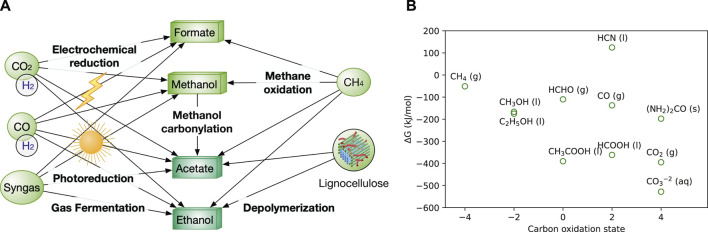
**(A)** Production network among various C1 and C2 feedstocks. CO_2_ and lignocellulosic biomass serve as the two ultimate carbon sources for all the liquid feedstocks. H_2_ is used for the reduction of CO_2_ and CO. O_2_ is required for the oxidization of methane to produce methanol and formate. Gaseous feedstocks (CO_2_, CO, H_2_) are in circles, while liquid feedstocks (methanol, formate) are in boxes. The formats of below figures are the same in colour and shapes. **(B)** Free energy and the oxidation state of C1 and C2 species (Aresta et al., 2014). Synonyms: g, gas; l, liquid, s, solid; aq, aqueous.

As a bulk chemical, methanol is produced >100 million metric tons annually from natural gas, syngas, and hydrogenation of CO_2_ (Jiang et al., 2021) (Figure 1A). Methanol price ($150–300/ton) is generally lower than sugar ($300–400/ton). Currently, methanol is produced from syngas, which is obtained mainly from natural gas, also from crude oil and coal (Ott et al., 2012) (Table 1). Hence, methanol price is highly dependent on natural gas. Formate is currently less abundant than methanol, but the technology to synthesize formate is advancing rapidly. One promising method is the electrochemical and photoreduction of CO_2_ to formate (Figure 1A), of which the cost is now ∼$500/ton and can be potentially reduced to $200/ton with cheaper electricity. If realized, formate will be a feedstock competitive against glucose that is priced $300–400/ton (Yishai et al., 2016; Claassens et al., 2020). Furthermore, electrochemical, photochemical, and catalytic methods for formate production that are being developed should drive up its availability.

C2 feedstocks are mainly on ethanol and acetate. Both are bulk chemicals. Ethanol is affordable, with a price of $250–350/ton. However, ethanol is mainly produced from starch-based resource, e.g., corn, which competes with food applications and loses 1/3 of carbon as CO_2_ (Table 1). Although ethanol production from lignocellulose still faces technical challenges and the U.S. Cellulosic Ethanol Industry has dwindled sharply (Lam et al., 2021), technological advance can reinvigorate the industry especially by reducing the overall cost especially from lignocellulose pretreatment. Acetate is currently produced from carbonylation of methanol (the main route) and sugar fermentation (used in food applications) (Table 1). In addition, new technologies are emerging, such as the gas-fermentation route to produce acetate using acetogenic microbes from C1 gas and hydrogen (H_2_), microbial electrosynthesis from CO_2_, lignocellulose depolymerization and anaerobic oxidation of methane by methanotrophs (Figure 1A). The current and potential alternative routes for acetate production have been discussed in detail previously (Kiefer et al., 2020). The current price of acetate ($300–450/ton) is considerably higher than that of ethanol and C1 feedstocks. Nevertheless, it is comparable to that of glucose (Table 1).

We have summarized the key information of NGFs in Table 1 and Figure 1B, including their sources, current prices, water solubility, Gibbs free energy, advantages, and disadvantages as biotechnological feedstocks. Of note, the price of bulk chemicals (methane, methanol, acetate and sugars) increases very rapidly in the past 6 months as the price of crude oil and natural gas surges caused by global supply chain disruption. Here, we use the median price in the past 5 years in Table 1. C2 feedstocks can be derived by C1 feedstocks by chemical and biological methods. Ultimately, CO_2_ can serve as the primary carbon source to produce all the liquid NGFs powered by electricity, sunlight, and inorganic electron donors (e.g., H_2,_ CO, sulphur) *via* either chemical synthesis (e.g., CO_2_ hydrogenation), biological routes (e.g., gas fermentation by acetogens) and their combination (e.g., microbial electrosynthesis) (Figure 1A). Despite huge technological challenges, most methods are close to industrial production or already commercialized. Methane oxidation can yield methanol, ethanol and even acetate, but is currently still premature (Shan et al., 2017; Xie et al., 2021). In addition, acetate is mainly produced from methanol carbonylation commercialized by BP chemicals and BASF. Lastly, in addition to CO_2_, the lignocellulosic biomass serves as another important feedstock due to its enormous quantities (Zhang and Too, 2019), and it has been extensively explored to produce ethanol by saccharification/fermentation (Lam et al., 2021) and acetate by pyrolysis and hydrolysis (Kiefer et al., 2020).

## Metabolic Engineering of Microbes to Use Next-Generation Feedstocks

Microorganisms have evolved amazing abilities to use NGFs as their main carbon sources. Autotrophic microbes can efficiently fix CO_2_ from the environment (Claassens et al., 2016); methanotrophs use methane actively (Strong et al., 2015); methylotrophs accept various reduced C1 substrates (such as methane, methanol, and other methylated compounds) as their sole sources of carbon and energy (Chistoserdova et al., 2009). Metabolic engineers have been learning from natural microbes by studying the molecular basis of NGF metabolism, key enzymes, cofactors required and the regulation of metabolic pathways. Of note, aerobic single carbon usage was extensively studied notably in 1970s when the pathways and physiology of these strains were established. In 1980s, the first group of anaerobes were characterized (Pacaud et al., 1986). However, the wealth of knowledge has not been used in industrial applications as methanol costs shut down a lot of this research after the first oil crisis. The syngas revolution as a product of waste stream disposal is creating a second-generation boom. Today, these findings inspire bioengineers to design synthetic microbes or to evolve natural or synthetic microbes that can utilize NGFs better and faster. In general, two strategies are adopted: 1) applying natural NGF-using microbes to convert NGFs to biomass and value-added products; 2) engineering synthetic microbes by transplanting the assimilation pathways into industrial workhorse microbes (e.g., *E. coli* and yeasts). Both strategies have advantages and disadvantages. The former requires to develop genetic engineering tools which are often very limited or even unavailable (Bourgade et al., 2021). In addition, low transformation efficiency (the efficiency of introducing extracellular DNA into microbial cells) strikingly hinder the progress of genetic engineering (Claassens et al., 2016; Jiang et al., 2021). Also, natural microbes have a restricted product spectrum. Hence, new pathways are required to diversify products (Cotton et al., 2020). Lastly, natural microorganisms have evolved their metabolic pathways and biosystems to produce biomass in natural environments. Therefore, major adaptions are required to allow these microbes to overproduce chemicals under industrial conditions. In contrast, industrial model microbes grow faster, support high cell density, have well established biosynthetic pathways to various value-added products, and more importantly, have advanced genetic engineering tools that can greatly accelerate strain engineering. However, as we will discuss in the following sections, the grafting of NGF assimilation pathway is not an easy task. Here, our focus is on the latter strategy on how to equip model microbes with the capability to use NGFs that are unnatural or unfavorable feedstocks.

### Natural Pathways for CO_2_, CO and Formate

Autotrophic organisms are able to fix CO_2_ (occasionally CO as well) from the environment using various pathways: 1) the Calvin-Benson-Bassham (CBB) cycle, 2) the Wood-Ljungdahl pathway (WLP), 3) the reductive tricarboxylic acid (rTCA) cycle, 4) the 3-hydroxypropionate–4-hydroxybutyrate (3HP-4HB) cycle, 5) the dicarboxylate–4-hydroxybutyrate (DC–4HB) cycle, 6) the 3-Hydroxypropionate (3HP) bicycle and 7) the reductive glycine pathway (rGlyP) (Claassens et al., 2016; Sánchez-Andrea et al., 2020). Among the seven pathways, formate can serve as the feedstock in the WLP and rGlyP instead of CO_2_ and H_2_.

**FIGURE 2:**
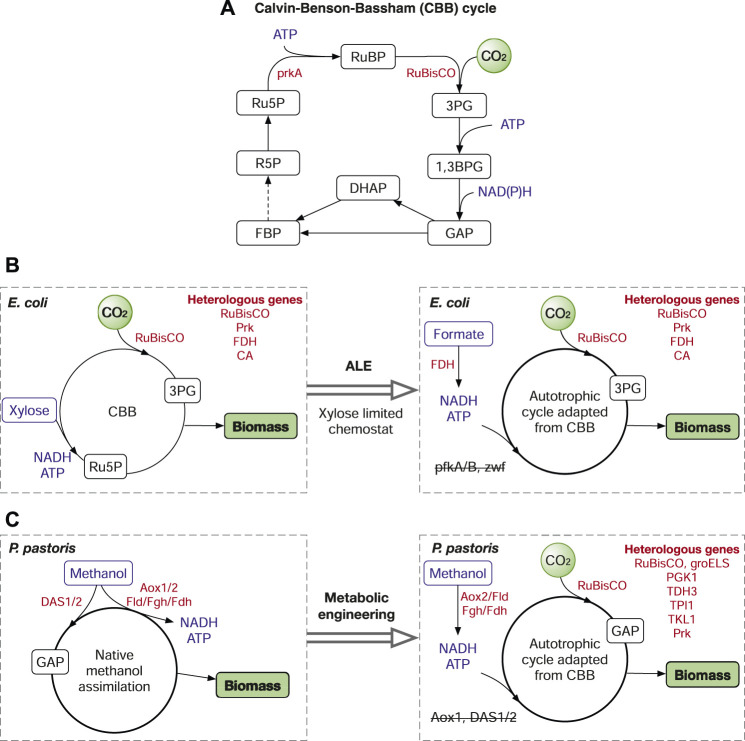
The Calvin-Benson-Bassham (CBB) cycle and its application in metabolic engineering. **(A)** The simplified CBB cycle. **(B)** Autotrophic *E. coli* harnessing the CBB cycle. **(C)** The CBB-enabled synthetic autotrophic *P. pastoris*. Enzymes: RuBisCO, ribulose-1,5-bisphosphate carboxylase/oxygenase; PrkA or Prk, phosphoribulokinase; FDH, formate dehydrogenase; CA, carbonic anhydrase; PfkA/B, 6-phosphofructokinase; Zwf, glucose-6-phosphate dehydrogenase; Aox1/2, alcohol oxidase; DAS1/2, dihydroxyacetone synthase; Fld1, formaldehyde dehydrogenase; Fgh, S-formylglutathione hydrolase; PGK1, phosphoglycerate kinase; TDH3, glyceraldehyde-3-phosphate dehydrogenase; TPI1, triosephosphate isomerase; TKL1, transketolose. Metabolites: RuBP, ribulose-1,5-bisphosphate; Ru5P, ribulose-5-phosphate; 3PG, 3-phosphoglycerate; 1,3BPG, 1,3-diphosphoglycerate; GAP, glyceraldehyde-3-phosphate; DHAP, dihydroxyacetone phosphate; FBP, fructose 1,6-bisphosphatase; R5P, ribose 5-phosphate. Reduced feedstocks (methanol, formate, xylose) and cofactors (ATP, NAD(P)H) are in blue. Genes/enzymes are in red.

The CBB cycle is ubiquitous in photoautotrophic organisms including plants, algae, and cyanobacteria and in some chemoautotrophic bacteria. The CO_2_ fixation in the CBB cycle depends on two key enzymes ribulose-1,5-bisphosphate carboxylase/oxygenase (RuBisCO) and phosphoribulokinase (PrkA), both of which are missing in heterotrophs (Figure 2A). To date, many studies have been reported on transplanting the CBB cycle into various heterotrophic microorganisms to fix CO_2_. The introduction of RuBisCo and PrkA into *E. coli* (Parikh et al., 2006) and *S. cerevisiae* (Guadalupe-Medina et al., 2013) enabled carboxylation of C5 sugars, which also increased the yield of glucose-to-ethanol fermentation in *S. cerevisiae*. Recently, a completely functional CBB cycle has been established in *E. coli* (Gleizer et al., 2019) with the introduction of RuBisCo, PrkA, carbonic anhydrase (CA) and formate dehydrogenase (FDH). In the study, formate is used as the energy source (Figure 2B). Of note, in addition to metabolic engineering, the successful transition of *E. coli* from a heterotroph to an autotroph was also attributed to laboratory evolution by gradually reducing the xylose supply in chemostat. Similarly, the CBB cycle has also been introduced into *Pichia pastoris*, which converted the methylotrophic yeast to an autotrophic yeast using methanol as the reducing power (Gassler et al., 2020). Unlike the *E. coli* study, the success of the *Pichia* study was mainly achieved by metabolic engineering, with the overexpression of eight genes, including six pathway genes (RuBisCo; Prk; PGK1, phosphoglycerate kinase; TDH3, glyceraldehyde-3-phosphate dehydrogenase; TPI1, triosephosphate isomerase; TKL1, transketolase) and two chaperone proteins groEL/S from *E. coli*, and the deletion of three genes, alcohol oxidase (Aox1) and two dihydroxyacetone synthases (DAS1/2) (Figure 2C). The deletion of Aox1 was to reduce the formation rate of formaldehyde, which was still produced by Aox2, a less active oxidase than Aox1. DAS1/2 are responsible for the conversion of methanol to central metabolites (GAP and dihydroxyacetone, or DHA), the deletion of DAS1/2 prevented methanol assimilation into biomass and made methanol only an energy source to supply ATP and NADH (Gassler et al., 2020). In addition, the CBB enzymes were introduced into the peroxisome, and the resulting strain was more efficient on CO_2_ fixation than that used cytosolic CBB pathway.

**FIGURE 3:**
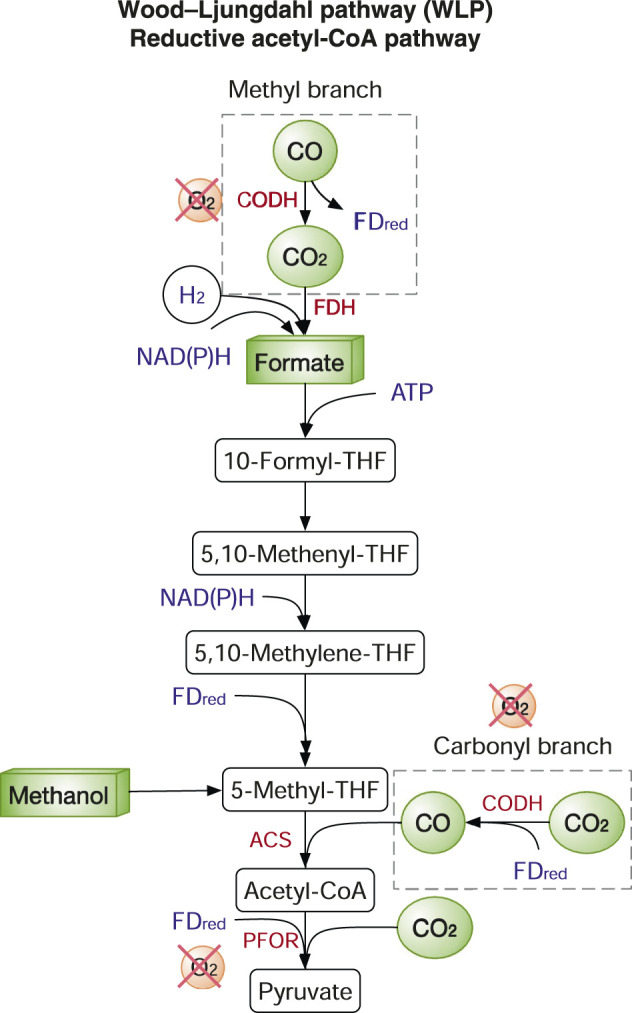
The Wood-Ljungdahl pathway (WLP). Enzymes: ACS, acetyl-CoA synthase; CODH, carbon monoxide dehydrogenase; FDH, ormatedehydrogenase; PFOR, pyruvate:ferredoxin oxidoreductase. Metabolites/cofactors: THF, tetrahydrofolate; FD_red_, reduced ferredoxins. Reduced cofactors (ATP, NAD(P)H, FD_red_) are in blue. Genes/enzymes are in red.

The WLP, also known as the reductive acetyl-coenzyme A (CoA) pathway, is the most efficient non-photosynthetic carbon fixation system. WLP requires only one ATP molecule per pyruvate. In contrast, the CBB cycle consumes seven ATP per pyruvate (Claassens et al., 2016). The key enzymes in the WLP are carbon monoxide dehydrogenase (CODH), formate dehydrogenase (FDH), acetyl-CoA synthase (ACS), pyruvate:ferredoxin oxidoreductase (PFOR) (Figure 3). CO_2_ is fixed by FDH to formate in the methyl branch, while CO used by ACS is in the carbonyl branch (Figure 3). As two key enzymes PFOR and CODH are oxygen sensitive, the WLP only functions anaerobically and so do the microbes harnessing the WLP, e.g., acetogens. Such anaerobes have extremely good carbon conversion efficiency but very poor growth as the production of acetate from acetyl-CoA is the principal ATP generating step. Nevertheless, acetogens have been widely explored both academically and industrially on gas fermentation using syngas and industrial waste gas to produce various chemicals such as acetate, ethanol, and butyrate. The topic has been extensively reviewed previously (Liew et al., 2016). Although the WLP is energetically highly efficient, reconstructing WLP in heterologous hosts is very challenging. The first attempt to transplant the WLP in *E. coli* failed as CODH and corrinoid iron-sulphur-containing protein (CoFESP) did not function well. Another recent study also failed to demonstrate strain growth on CO and H_2_ (Liew et al., 2016). One major difficulty is that *E. coli* and yeasts lack the proper intracellular conditions especially on the cofactor production (e.g., vitamin B12) and assembly of delicate metal centres.

**FIGURE 4:**
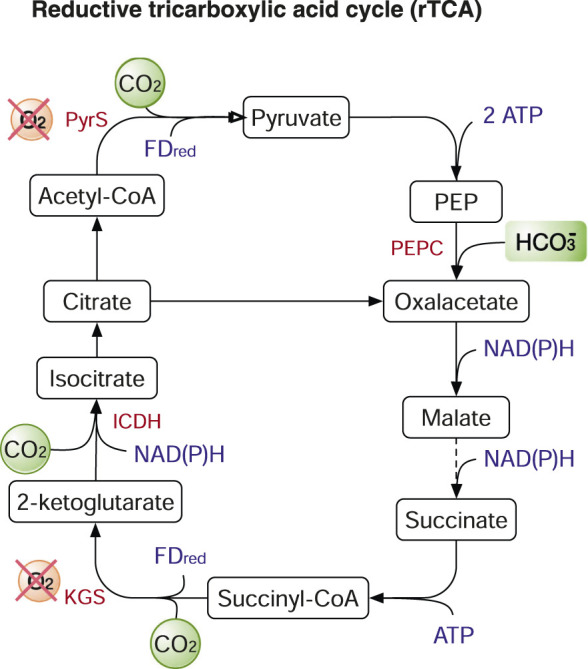
The reductive tricarboxylic acid (rTCA) cycle. Enzymes: PyrS, pyruvate synthase; PEPC, PEP carboxylase; KGS, α-ketoglutarate synthase; ICDH, isocitrate dehydrogenase. Metabolites: PEP, phosphoenolpyruvate. Dashed arrows are multiple enzymatic reactions.

The rTCA cycle, identical to the TCA cycle but in the reverse (reductive) direction (Figure 4), depends on four key enzymes to fix CO_2_: α-ketoglutarate synthase (KGS) or α-ketoglutarate:ferredoxin oxidoreductase; isocitrate dehydrogenase (ICDH); phosphoenolpyruvate (PEP) carboxylase (PEPC); pyruvate synthase (PyrS). Among the four enzymes, KGS and PyrS are oxygen sensitive and require ferredoxin as the reducing cofactor. ICDH uses NAD(P)H as the reducing cofactor. PEPC, which prefers bicarbonate than CO_2_ as the substrate, is also the key enzyme in C4 carbon fixation or the Hatch–Slack pathway, which contributes to reduce the wasteful process of photorespiration of RuBisCO in C4 plants (Paulus et al., 2013). As one of the most energy-efficient carbon fixation pathways, the rTCA cycle (requiring 1-2 ATP per pyruvate) has been proposed to produce chemicals and fuels from atmospheric CO_2_ using microbial cells. This process is named the third-generation biorefineries (Liu et al., 2020). However, to date, few studies have applied the rTCA cycle in metabolic engineering for carbon fixation. In a recent study, rTCA cycle was used to recycle CO_2_ in *E. coli* using KGS. The CO_2_ production was reduced. Concurrently, formate production was observed, and the production of acetate and ethanol were increased (Chen C.-H. et al., 2020).

**FIGURE 5:**
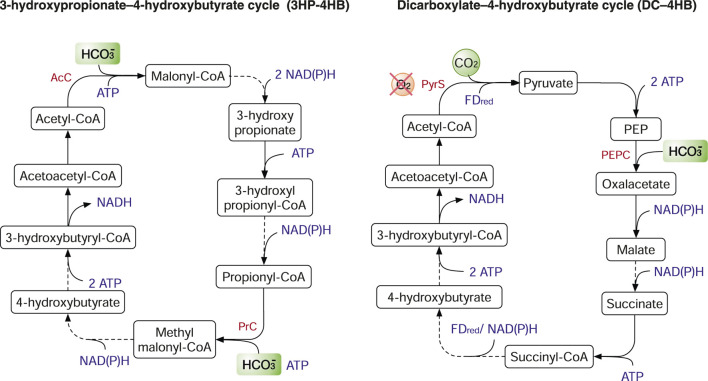
The 3-hydroxypropionate–4-hydroxybutyrate (3HP-4HB) cycle and the dicarboxylate–4-hydroxybutyrate (DC–4HB) cycle. Enzymes: AcC, acetyl-CoA carboxylase; PrC, propionyl-CoA carboxylase; PyrS, pyruvate synthase; PEPC, PEP carboxylase.

The 3HP-4HB cycle depends on two enzymes to fix CO_2_, acetyl-CoA carboxylase (AcC) and propionyl-CoA carboxylase (PrC). AcC and PrC catalyse the addition of bicarbonate to acetyl-CoA and propionyl-CoA, respectively (Figure 5). The 3HP-4HB cycle has two variants of different energy efficiency. In the *Crenarchaeota* phylum, the 3HP-4HB cycle requires 9–10 ATP per pyruvate. In contrast, the 3HP-4HB cycle in the *Thaumarchaeota* pythlum requires only five ATP per pyruvate (Claassens et al., 2016). Like the rTCA cycle, the DC–4HB cycle also uses PyrS and PEPC to fix CO_2_. As PyrS is oxygen sensitive, microbes such those in the order *Thermoproteales* using the DC–4HB cycle typically grow anaerobically (Hawkins et al., 2013). In the 3HP-4HB and DC-4HB cycles, two CO_2_ molecules are fixed by PyrS to acetyl-CoA (C2) to produce succinyl-CoA (C4). Subsequently, succinyl-CoA is rearranged to acetoacetyl-CoA that is cleaved into two molecules of acetyl-CoA. In contrast, the acetyl-CoA is re-generated from citrate in the rTCA cycle (Figure 4). The 3HP bicycle is energetically expensive as it consumes seven ATP per pyruvate. The 3HP bicycle shares half the metabolic pathway (from malonlyl-CoA to succinyl-CoA) as that of the 3HP-4HB cycle and the two key enzymes for CO_2_ fixation, AcC and PrC. However, the re-generation of acetyl-CoA in the 3HP bicycle has two routes: 1) from malyl-CoA that is produced from malate; 2) from citramalyl-CoA that is synthesized from glyoxylate and propionyl-CoA (Herter et al., 2002; Mattozzi et al., 2013). To date, the metabolic engineering applications of the 3HP-4HB cycle on carbon fixation are scarce. To our knowledge, the only example is that 3HP-4HB cycle genes are partially transplanted into the thermophilic host *Pyrococcus furiosus* to produce 3-hydroxypropionate from CO_2_ and H_2_ (Keller et al., 2013). The DC-4HB cycle has not been expressed in industrially relevant microbes for CO_2_ fixation. Yet, the 3HP bicycle has been divided into four sub-pathways and individually expressed in *E. coli* (Mattozzi et al., 2013). Although all the sub-pathways were functional, none of them could support autotrophic growth, and the attempt to reconstruct the complete 3HP bicycle was not successful in *E. coli* (Claassens et al., 2016).

**FIGURE 6:**
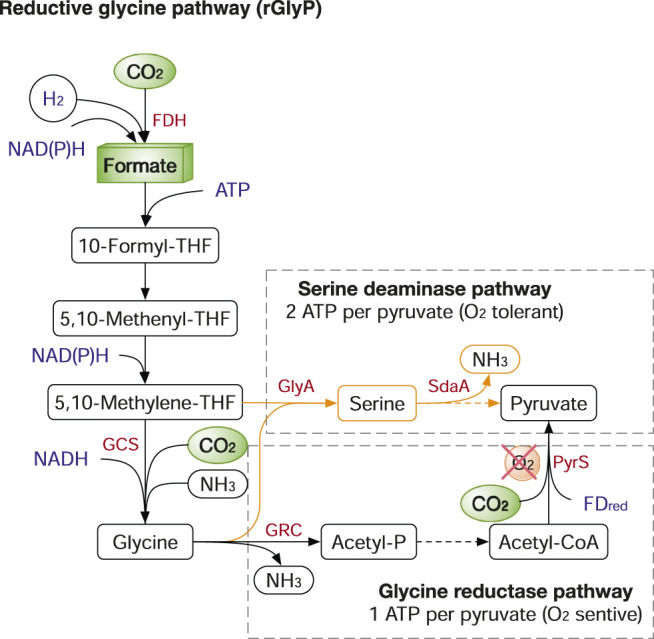
The reductive glycine pathway (rGlyP). The rGlyP has two variants: serine deaminase pathway (in orange) and glycine reductase pathway (in black). Enzymes: FDH, ormatedehydrogenase; GCS, glycine cleavage/synthase system; GlyA, serine hydroxymethyltransferase; Sda, serine deaminase; GRC, Glycine reductase complex. Metabolites: THF, tetrahydrofolate.

The rGlyP, naturally used in the sulphate-reducing bacterium *Desulfovibrio desulfuricans* (Sánchez-Andrea et al., 2020), is another energy-efficient carbon fixation system (1-2 ATP per pyruvate), almost matching the WJP. Also, the rGlyP shares half of its pathway with the WJP, from CO_2_/CO to 5,10-methylene-THF (Figure 6). Glycine cleavage/synthase system (GCS), a reversible four-component enzyme, is the key enzyme for CO_2_ fixation and catalyses 5,10-methylene-THF, CO_2_ and NH_3_ to produce glycine. Glycine can be further assimilated to pyruvate and biomass via two main routes: 1) aerobically, by the serine deaminase pathway variant, which consumes two ATP per pyruvate; 2) anaerobically, by the glycine reductase pathway variant, which requires one ATP per pyruvate (Figure 6). The structural simplicity, energy efficiency, and the ability to operate both aerobically and anaerobically make rGlyP a very attractive route for metabolic engineering in both CO_2_ fixation and formate assimilation. Recently, the rGlyP has been widely reconstructed in various microbes and tested in both aerobic and anaerobic conditions. In 2018, Arren Bar-Even group first assembled the synthetic rGlyP in *E. coli* to produce serine from formate and CO_2_ (Yishai et al., 2018), and further engineered *E. coli* to grow on formate and CO_2_ and achieved relatively fast growth with 8 h doubling time after ALE from the initial doubling time of ∼70 h (Kim et al., 2020). The core rGlyP has also been functionally expressed in *S. cerevisiae* to produce glycine from formate and CO_2_ (Cruz et al., 2019). The rGlyP is also explored to replace the native Calvin cycle in *Cupriavidus necator* to grow on formate, achieved 2.6 g CDW/mole-formate (Claassens, 2021). Also, the GCS is successfully introduced into *Clostridium pasteurianum* for anaerobic utilization of formate and CO_2_ (Hong et al., 2021).

The CO_2_ fixation pathways have different energy efficiencies. Typically, the pathways operating in anaerobic conditions have higher ATP efficiency: the WLP and rGlyP require only 0.5 ATP per CO_2_ fixed in anaerobic conditions. In contrast, those operating in aerobic and microaerobic conditions require more ATP: the CBB cycle, 3 ATP/CO_2_; rTCA cycle, 1 ATP/CO_2_; the 3HP-4HB cycle, 2 ATP/CO_2_; the DC–4HB cycle, 1.5 ATP/CO_2_; the 3HP bicycle, 1.67 ATP/CO_2_. The ATP efficiency can better support the growth of anaerobes in which relatively less ATP is available than that in aerobes. In addition to ATP, the requirement of NAD(P)H equivalents is the same for various pathways at 2 NAD(P)H/CO_2_, assuming the product is acetyl-CoA (if pyruvate were the product, it would require 1.67 NAD(P)H/CO_2_) (Xiao et al., 2022). Also, different enzymes may use different reducing equivalents and electron donor. For example, CODH and PFOR in the WLP, as well as PyrS and KGS in the rTCA cycle, require ferredoxin as cofactors; other enzymes mostly use NAD(P)H as the reducing equivalents (Figures 2–6). Of note, ferredoxin (*E* ^′0^ = –430 mV) has a higher energetic driving force than NAD(P)H (*E* ^′0^ = –320 mV). This also contributes to the difference in energetic efficiency of various pathways.

### Natural Pathways for Methane and Methanol

The assimilation of methane in methanotrophs starts with its oxidation to methanol catalyzed by methane monooxygenases (MMOs). Two types of MMO are identified in methanotrophs: the intracellular soluble form (sMMO) present in several methanotrophs; and the more ubiquitous membrane-bound, particulate enzyme complex (pMMO) (Hakemian and Rosenzweig, 2007). sMMO has wider substrate specificity but can be inhibited by high copper concentration. Owing to the membrane association, pMMO has greater access to methane than sMMO and is proposed to oxidize methane more quickly (Ge et al., 2014). It is very challenging to express functional sMMOs or pMMOs in *E. coli*. Many attempts have made with limited success (Balasubramanian et al., 2010; Kim et al., 2019). A recent study developed a pMMO-mimetic catalytic protein by assembling the catalytic domains of pMMO on apoferritin as a biosynthetic scaffold. The pMMO-mimetic enzyme has a turnover number (0.084 s^−1^) comparable to that of native pMMO and can be produced in *E. coli* with a high yield (Kim et al., 2019), paving the way for future metabolic engineering applications on methane.

**FIGURE 7:**
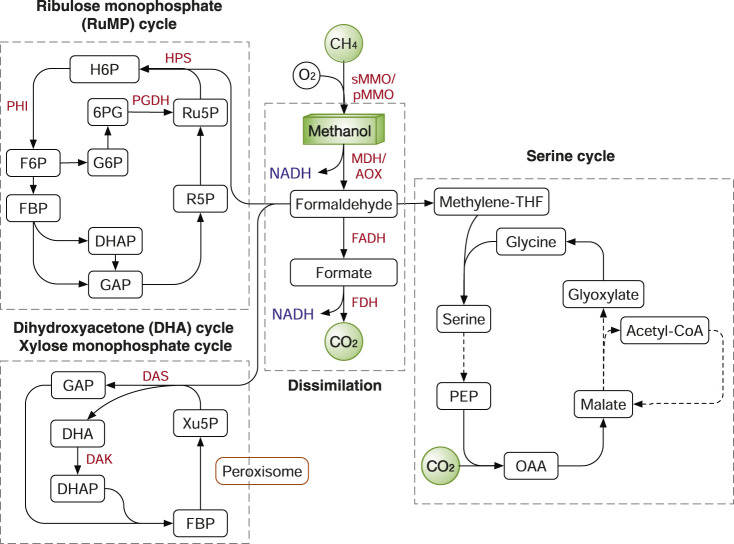
Methane and methanol assimilation routes. In nature, three main routes were identified: ribulose monophosphate (RuMP) cycle, xylose monophosphate (XuMP) cycle or dihydroxyacetone (DHA) cycle and Serine cycle. Enzymes: HPS, 3-hexulose-6-phosphate synthase; PHI, 6-phosphate-3-hexuloisomerase; PGDH, 6-phosphogluconate dehydrogenase; MMO, methane monooxygenase; MDH, methanol dehydrogenase; AOX, alcohol oxidase; FADH, formaldehyde dehydrogenase; FDH, formate dehydrogenase. Metabolites: H6P, hexulose 6-phosphate; F6P, fructose-6-phosphate; FBP, fructose 1,6-bisphosphatase; G6P, glucose-6-phosphate; 6PG, 6-phosphogluconate; GAP, glyceraldehyde-3-phosphate; DHAP, dihydroxyacetone phosphate; R5P, ribose 5-phosphate; DHA, dihydroxyacetone; Xu5P, xylulose-5-phosphate; PEP, phosphoenolpyruvate; OAA, oxaloacetate.

After the methane oxidation step, its pathway may merge with the methanol assimilation pathway. Firstly, methanol is oxidized into formaldehyde by methanol dehydrogenase (MDH) in prokaryotes or by alcohol oxidase (AOX) in yeasts (Pfeifenschneider et al., 2017) (Figure 7). In methylotrophic bacteria such as *Bacillus methanolicus* and *Methylobacterium extorquens*, MDH oxidizes methanol using pyrroloquinoline quinone (PQQ) or nicotinamide adenine dinucleotide (NAD+) as dependent electron acceptors (Pfeifenschneider et al., 2017). In methylotrophic yeasts such as *Candida boidinii*, *Pichia pastoris* and *Hansenula polymorpha*, methanol is oxidized by AOX with molecular oxygen as an electron acceptor (Yurimoto and Sakai, 2019).

Formaldehyde is either oxidized to CO_2_ for NADH production or assimilated into biomass. The breakdown of formaldehyde to CO_2_ is catalysed by the two enzymes formaldehyde dehydrogenase (FADH) and formate dehydrogenase (FDH) (Figure 7). The dissimilation process not only supplies NADH and also serves as an important way to detoxify formaldehyde. In methanotrophs, formaldehyde is assimilated by mainly two routes: the ribulose monophosphate (RuMP) cycle and the serine cycle (Gęsicka et al., 2021). In methylotrophs, in addition to the two routes, additional pathways include: the Calvin cycle in the soil bacterium *Cupriavidus necator* and the dihydroxyacetone (DHA) cycle, also known as xylulose monophosphate (XuMP) pathway, in methylotrophic yeasts. In addition, as we previously discussed, the WLP and rGlyP can also be used to assimilate methanol and formate (Kim et al., 2020) (Figures 3, 6).

The RuMP cycle has several variants with different energetic efficiency as described and compared previously (Cotton et al., 2020). Nevertheless, all the RuMP cycle variants share the common enzymes 3-hexulose-6-phosphate synthase (HPS) and 6-phosphate-3-hexuloisomerase (PHI) (Figure 7). In different prokaryotes, F6P is channelled into various metabolic pathways to regenerate Ru5P, including glycolysis, non-oxidative pentose phosphate pathway (PPP), the Entner–Doudoroff (ED) pathway or the pathway via sedoheptulose 7-phosphate (Cotton et al., 2020). In the DHA (XuMP) cycle, formaldehyde and xylulose 5-phosphate (Xu5P; C5 compound) are catalysed by dihydroxyacetone synthase (DAS) to generate GAP and DHA, which are further channelled back to regenerate Xu5P (Figure 7). Different from bacteria, the methylotrophic yeasts express the methanol assimilation enzymes in the organelle peroxisome. The compartmentalization in peroxisomes can isolate the toxic intermediates (e.g., formaldehyde) from the rest of cells and is potentially more efficient than prokaryote systems.

The RuMP cycle shares most enzymes with sugar metabolism in sugar heterotrophs such as *E. coli*, except for three enzymes: MDH, HPS, PHI. However, simple expression of the three enzymes is insufficient to convert *E. coli* to a methylotroph growing solely on methanol, although methanol could be assimilated into the central metabolism with the supplementation of xylose (Müller et al., 2015), glucose (Bennett et al., 2018) or threonine (Gonzalez et al., 2018) as co-substrate. The critical issue is how to elegantly balance the generation and consumption of formaldehyde and preventing its accumulation intracellularly. The accumulation of formaldehyde induces DNA-protein crosslinking that leads to cell death (Chen F. Y.-H. et al., 2020). Recently, this problem has been solved by rational pathway design and ALE. Kinetic modelling identified that high activities of phosphofructokinase (Pfk) and glyceraldehyde 3-phosphate dehydrogenase (Gapdh) may destabilize the metabolic system by diverting the flux away from the RuMP cycle. After the deletion of the two genes, the *E. coli* strain carrying the RuMP cycle genes was evolved to grow solely on methanol and higher growth rate was achieved with a doubling time of 8 h (Chen F. Y.-H. et al., 2020). The growth rate of the evolved *E. coli* strain is comparable to the *E. coli* using the rGlyP as we discussed previously (Kim et al., 2020). Also, *Corynebacterium glutamicum* is explored for methanol assimilation (Zhang B. et al., 2020).

*S. cerevisiae*, a non-methylotrophic yeast, has also been studied to assimilate methanol. A recent study proved that the *S. cerevisiae* CEN. PK strain has the native methanol assimilation capability using ^13^C tracer-analysis (Espinosa et al., 2020). ALE and sequencing experiments further pinpointed an uncharacterized transcriptional regulator Ygr067cp that supports improved methanol assimilation. It was found that the deletion of the alcohol dehydrogenase 2 (*adh2*) or the acetyl-CoA synthetase gene (*acs1*) reduced methanol assimilation. However, the key enzymes for methanol assimilation in *S. cerevisiae* are still unknown. In another study, the transplantation of the DHA cycle genes from *P. pastoris* supported *S. cerevisiae* growth on methanol (Dai et al., 2017). Also, another non-methylotrophic yeast *Yarrowia lipolytica* has been recently engineered to assimilate methanol by introducing the DHA and RuMP cycle genes, as well as by using laboratory evolution (Wang et al., 2021).

### Synthetic Routes for C1 Feedstock Assimilation

It is straightforward to use wildtype microbes and to harness natural assimilation pathways. However, natural assimilation pathways are not always the best choice as they can be rather complex involving too many enzymes (e.g., 3-HP bicycle requires 18 enzymes), kinetically inefficient due to inefficient/unspecific enzymes (e.g., RuBisCO catalyses side reactions with O2 that under atmospheric conditions), have tight intrinsic regulations (e.g., NADH/NAD^+^ regulation) or contain special cofactors (e.g., the WLP) that are hard to reconstitute in industrial workhorse strains. Therefore, it is also attractive to design synthetic routes using novel enzymes and novel combinations of well-studied enzymes. Well-designed synthetic routes can be superior to natural ones in: 1) circumventing the limitations of natural pathways, for example, the use of oxygen-tolerant enzymes makes rGlyP also functional in aerobic conditions (Bang and Lee, 2018; Bang et al., 2020); 2) simplifying the metabolic pathway using less enzymes (Xiao et al., 2022); 3) bypassing the host metabolism and regulations using orthogonal designs (Chou et al., 2021); 4) achieving higher efficiency (Schwander et al., 2016; Cai et al., 2021) or making the pathway more thermodynamically favourable (Siegel et al., 2015).

**FIGURE 8:**
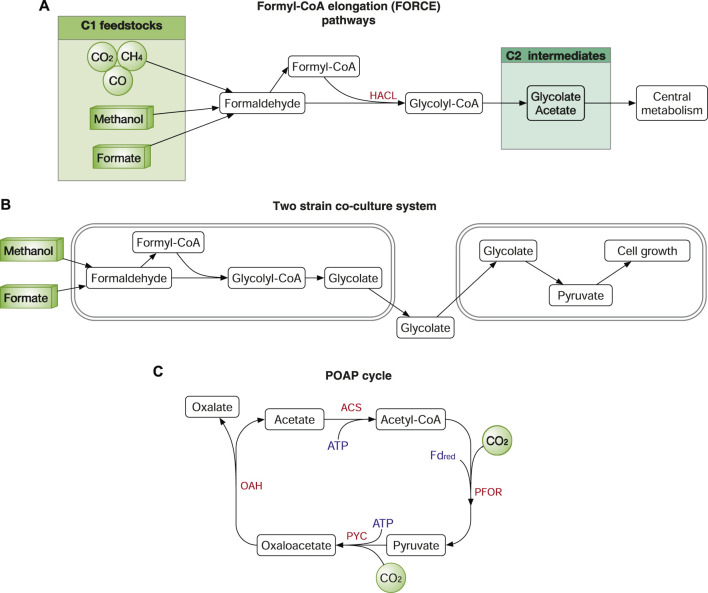
Synthetic routes for C1-feedstock assimilation. **(A)** Synthetic routes of the formyl-CoA elongation (FORCE) pathways. **(B)** Two strain co-culture system using the FORCE pathways. **(C)** A synthetic CO_2_ fixation pathway, the POAP cycle. Enzymes: HACL, 2-hydroxyacyl-CoA lyase; PYC, pyruvate carboxylase; OAH, oxaloacetate acetylhydrolase; ACS, acetate-CoA ligase, and PFOR, pyruvate synthase. Cofactors: FD_red_, reduced ferredoxins.

Formolase, a computationally designed enzyme that catalyses formaldehyde to produce DHA, supports a synthetic pathway that assimilates formate to DHAP and further into central metabolism (Siegel et al., 2015). Another interesting enzyme is 2-hydroxyacyl-CoA lyase (HACL) that catalyses the ligation of carbonyl-containing molecules of different chain lengths with formyl-CoA to produce C1-elongated 2-hydroxyacyl-CoAs (Chou et al., 2019). HACL enables the bioconversion C1 feedstock to C2 or longer products, such as glycolate and 2-hydroxyisobutyrate. Using HACL, the same team further developed the formyl-CoA elongation (FORCE) pathways, which can use various C1 feedstocks (CO_2_, CO, formate, formaldehyde, methanol, and methane) to produce multi-carbon products including glycolate, ethylene glycol, ethanol and glycerate (Figure 8A). The FORCE system also demonstrated the potential in converting *E. coli* to synthetic methylotrophy in two-strain co-culture system (Chou et al., 2021). In the co-culture system, the first *E. coli* strain converts the C1 feedstock formate or methanol to the C2 product glycolate, which is further used as the feedstock to support the growth of the second *E. coli* strain (Figure 8B).

Another example of a synthetic CO_2_ fixation pathway is designed by metabolic retrosynthesis. The synthetic pathway is named the crotonyl–coenzyme A (CoA)/ethylmalonyl-CoA/hydroxybutyryl-CoA (CETCH) cycle, comprising a reaction network of 17 enzymes from nine different organisms (Schwander et al., 2016). Using the more efficient enoyl-CoA carboxylases/reductases (ECRs), the CETCH cycle is about 2–4 times more efficient than that of RuBisCO. In a very recent study, a minimized synthetic CO_2_ fixation cycle was designed and optimized with only four enzymes (Xiao et al., 2022). The synthetic cycle, named the POAP cycle, consists of pyruvate carboxylase, oxaloacetate acetylhydrolase, acetate-CoA ligase, and pyruvate synthase (Figure 8C). According to the study (Xiao et al., 2022), the POAP cycle fixes two CO_2_ to produce oxalate using two ATP and one NAD(P)H, which is energetically more efficient than the seven natural and one synthetic CO_2_ fixation pathways, the CBB, 3HP-4HB, rTCA, DC-4HB, 3HP and CETCH cycle, rGlyP and the WLP. However, POAP has yet to be demonstrated in microbes, and the *in vivo* reconstitution might face some challenges, e.g., the conversion from pyruvate and CO_2_ to oxaloacetate can be outcompeted by other native pathways.

### Natural Pathways for Ethanol and Acetate Assimilation

As compared to C1 feedstocks, the assimilation of C2 feedstocks by microorganisms is considerably easier and more efficient (Ma et al., 2022). Industrial model microbes are naturally, or with minimal engineering, capable of using both acetate and ethanol efficiently. Therefore, the current focus of C2 feedstocks is not on how to produce biomass but on how to better use them to produce valuable products in high yields and productivities.

Acetate primarily enters microbial cells by passive diffusion in the form of undissociated molecules (HAc). In addition, some bacteria (e.g., *E. coli* and *Corynebacterium glutamicum*) also use transporters for the uptake of dissociated acetate molecules (Ac^−^) (Gimenez et al., 2003; Sá-Pessoa et al., 2013). The assimilation of acetate requires only one or two enzymes. In *E. coli* and *C. glutamicum*, the acetate is assimilated by two routes: 1) ACS, which converts acetate to acetyl-CoA; 2) AckA and Ptak, which together catalyse acetate to acetyl-phosphate and further to produce acetyl-CoA (Enjalbert et al., 2017). Both routes require one ATP per acetyl-CoA. In some bacteria such as *Pseudomonas* sp. and acetic acid bacteria, the acetate assimilation is catalysed by succinylCoA:acetate CoA-transferase (SCACT). SCACT coverts acetate to acetyl-CoA using succinyl-CoA as CoA donor (Mullins et al., 2008). In *S. cerevisiae*, ACS1 is responsible for the assimilation of acetate and ethanol. ACS1 is expressed in peroxisome together with the glyoxylate shunt enzymes (e.g., citrate synthase, malate synthase) (Berg and Steensma, 1995). As described in Table 1, acetyl-CoA is a precursor that leads to various value-added bioproducts. As such, acetate has been used to produce many high-value products including small organic acids, alcohols, amino acids, terpenoids and lipids in several model microbes (Kiefer et al., 2020; Kim et al., 2021). For example, *E. coli* has been engineered to produce succinic acid (Huang et al., 2018), mevalonic acid (Xu X. et al., 2017) and β-caryophyllene (Yang and Nie, 2016) with relatively good yields. The oleaginous yeasts *Rhodosporidium toruloides* and *Y. lipolytica* were explored to produce lipids solely from acetate (Huang et al., 2016; Xu J. et al., 2017). *Trichosporon cutaneum* AS 2.571 was reported to have >2× cell mass and >3× lipid productivity of *Y. lipolytica* AS 2.1398 using acetate as the sole carbon source. *R. toruloides* was found poor in using acetate (Xu J. et al., 2017), however, our recent work showed that some *R. toruloides* strains have similar cell mass productivity to *T. cutaneum* AS 2.571. With ALE approach, we have increased the growth rate of *R. toruloides* on acetate by >30% as compared to the wild type, also shortened the lag phase from >20 h in wildtype to ∼10 h (Manuscript in preparation). Using metabolic engineering strategies that we developed (Zhang et al., 2018; Zhang and Too, 2020), we are currently engineering *E. coli* to produce terpenoids from acetate.

The assimilation of ethanol has two main routes: 1) alcohol dehydrogenase and acetaldehyde dehydrogenase (acetylating) that converts ethanol to acetaldehyde further to acetyl-CoA; 2) the acetate route in eukaryotes such as *S. cerevisiae*, ethanol is first converted to acetate via acetaldehyde, and acetate is assimilated to acetyl-CoA. The first route is found mainly in bacteria such as *Clostridium acetobutylicum* and *E. coli*. In *S. cerevisiae*, ethanol is oxidized by the alcohol dehydrogenase (*adh2* or *adh4*) into acetaldehyde, which is further converted to acetate by aldehyde dehydrogenase (*ald4* and *ald5*). The conversion of ethanol to acetate generates two NADH, which can be used for ATP regeneration. Therefore, higher theoretical yields are expected from ethanol than from acetate for reduced products. For example, the theoretical production yield of poly(3-hydroxybutyrate) (PHB) from ethanol, acetate and glucose are 0.935, 0.51 and 0.478, respectively (Sun et al., 2020). However, the assimilation of ethanol is a heat generating and oxygen-intensive process that may severely increase the production cost (Westfall et al., 2012). The acetate route genes were introduced into *E. coli* to assimilate ethanol via acetate as the intermediate (Cao et al., 2020). In addition, *E. coli* has been engineered to grow efficiently on ethanol by optimizing the two enzymes acetaldehyde dehydrogenase and alcohol dehydrogenase (acetylating). The ethanol-assimilating *E. coli* strain was further engineered to produce PHB or prenol from ethanol (Liang et al., 2021). In addition, ethanol has been used as sole or co-substrate with glucose to produce artemisinin precursor in *S. cerevisiae* (Westfall et al., 2012) and to produce up to 138 g/L of biomass in *Candida brassicae* (Mori et al., 1979). Although ethanol has a good potential, to date, ethanol has not been well explored as feedstock in metabolic engineering.

## Bioprocess Development in Using Next-Generation Feedstocks

### Preparation of C1 and C2 Feedstocks for Microbial Fermentation

The selection of NGFs is crucial to establish efficient, rapid, and industry-compatible bioprocesses for the bioproduction of value-added chemicals. This requires a holistic view to fulfil the requirements of microorganisms (e.g., metabolic pathways, cofactors and growth conditions) and NGF-specific process design. Firstly, the continuous availability of NGF must be ensured and all the feedstocks chosen here fulfil this requirement. Indirect supply costs such as transport and purification must be evaluated, in which liquid NGFs are superior to gaseous NGFs (CO_2_, CO, and CH_4_). The safety profiles of the NGFs are directly related to this cost, as properties such as toxicity and flammability are key cost drivers, as summarized in Table 1. Next, special attention must be paid to the physical and chemical properties of the NGF, such as the aggregate state (gaseous or liquid) and water solubility, as these critically restrict mass transfer. Comparing next-generation gaseous and liquid feedstocks, their solubility differs by several orders of magnitude (Table 1). For comparison, the traditional feedstock glucose (909 g/L at 25°C) has a similar solubility to next-generation liquid feedstocks. This issue can be approached from different angles. Elevated pressure fermentation at 5–10 bar was identified as a straightforward but underexplored approach to increase mass transfer (Hecke et al., 2019). Beside the continuous stirred tank reactor that is typically used for liquid feedstocks, different fermentation systems have been tested such as bubble column, gas lift reactor, loop reactor, trickle bed reactor, membrane reactor and moving bed biofilm reactor amongst others which are discussed in detail elsewhere (Stoll et al., 2020). Another strategy is to convert gaseous feedstocks to liquid feedstocks using chemical (syngas to methanol), biological (gas fermentation to acetate) or hybrid approaches (microbial electrosynthesis), which are discussed in our previous section.

Another important factor is possible toxic effects of NGFs on the microorganism as well as possible adverse impacts of potential impurities (e.g., flue gas contains CO_2_ but may also contain sulfur dioxide). Excessive NGF concentrations and the presence of significant amounts of trace gases can slow down or even stop the growth of microorganisms, resulting in low productivities. This issue can be overcome by strain engineering (e.g., development of detoxification pathways) or by bioprocess development. Particularly, appropriate feeding strategies and, if necessary, NGF purification process can be optimised to minimize/remove those toxic impurities. Exemplarily for syngas, depending on the gasification material and conditions used, impurities such as H_2_O, N_2_, particulates, alkali compounds (e.g., KOH, KCl), tars (organic hydrocarbon compounds >78 g/mol), nitrogen compounds (e.g., ammonia, hydrogen cyanide, and NO_x_), sulfur compounds (e.g., H_2_S), carbon-oxygen-sufur compounds (COS), halogen compounds (e.g., HCl), and heavy metals (e.g., Zn, Pb, Cd) amongst others can be found (Martin and Wolfgang, 2009). As gas fermentation is anaerobic, even oxygen is considered a gas impurity and has to be removed beforehand either by catalytic (Yan et al., 2013) or biologic processes (Mohr et al., 2019). An example highlights the importance of feedstock pretreatment. In 2014, in a commercial setting to produce ethanol from syngas, the plant was inoperable due to undetected hydrogen cyanide. The plant went into operation after the removal of the impurity from the feedstock was achieved by an additional treatment step (https://www.biofuelsdigest.com/bdigest/2014/09/05/on-the-mend-why-ineos-bio-isnt-reporting-much-ethanol-production). In addition to feedstock toxicity, inhibitory effects of products should also be evaluated for all fermentation systems.

### Bioprocess Challenge and Optimization for Next-Generation Feedstock Utilization

**FIGURE 9:**
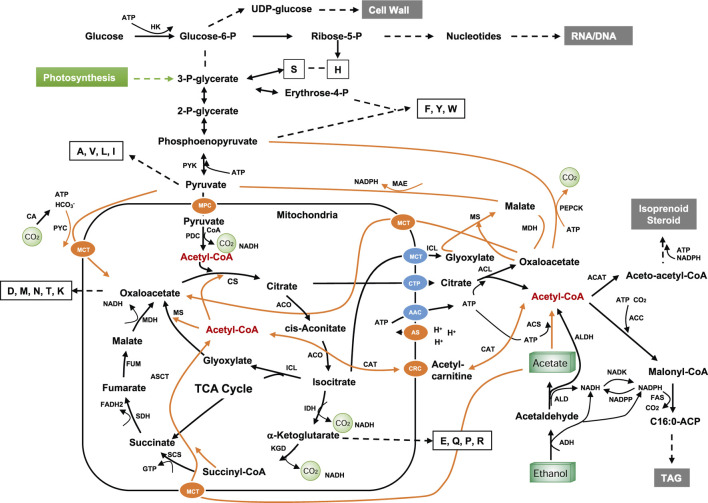
Metabolic pathway for C2 feedstock. Dotted lines indicate multiple steps; letters in empty boxes indicate the single letter codes for amino acids; orange lines indicate potential routes for C2 feedstock utilization; AAC, ADP/ATP carrier protein; ACC, acetyl-CoA carboxylase; ACAT, acetyl-CoA acetyltransferase; ADH, alcohol dehydrogenase; ALD, aldehyde dehydrogenase; ALDH, Acetaldehyde dehydrogenase (EC 1.2.1.10); ACO, aconitase; ACS, acetyl-CoA synthetase; ACL, ATP-citrate lyase; AS, ATP synthase; ASCT, acetate:succinate CoA-transferase; CA, carbonic anhydrase; CAT, carnitine acetyltransferase; CRC, carnitine carrier; CTP, mitochondria citrate transporter; CS, citrate synthase, FAS, fatty acid synthase; FUM, fumarase; HK, hexokinase; IDH, isocitrate dehydrogenase; ICL, isocitrate lyase; KDG, α-ketoglutarate dehydrogenase complex; MAE, malic enzyme; MDH, malate dehydrogenase; MPC, mitochondrial pyruvate carrier; MS, malate synthase; NADK, NAD+ kinase; NADPP, NADPH phosphatase; SCS, succinyl-CoA synthetase; SDH, succinate dehydrogenase; PDC, pyruvate dehydrogenase complex; PEPCK, Phosphoenolpyruvate carboxykinase; PYC, pyruvate carboxylase; PYK, pyruvate kinase; MPC, mitochondrial pyruvate carrier; MCT, monocarboxylate transporters. The feeding point to photosynthetic product in plants and algae is shown in green.

One of biggest challenges in using NGFs for microbial fermentation is the slow cell growth and low maximal cell density (biomass). This is due to: 1) the cytotoxicity and low energy content of the C1/C2 substrates (Figure 1B); 2) limited supply of amino acids, nucleotide sugar and acetyl-CoA that are the precursors for the biosynthetic machinery (such as ribosomes and enzymes), cell wall, cytoskeleton and membranes; 3) formaldehyde and acetaldehyde, the minor metabolic intermediate of methanol and ethanol respectively, are highly reactive and cause cell cycle arrest which is triggered by the modification or cross-linking nucleic acids and proteins (Chen F. Y.-H. et al., 2020). To reduce the cytotoxicity of C1/C2 feedstock, substrate loading has to be maintained low, making it more unfavorable for gluconeogenesis (the reverse reactions of glycolysis) which makes UDP-glucose, certain amino acids such as S, H, F, Y, W, A, V, L, I and acetyl-CoA (Figure 9) (pathway for C1 metabolism is not discussed here as it can be found in many review papers). Thus, supplementation of medium with yeast extract, certain amino acids and vitamins has great impact on cell growth and final metabolite yields (Kaushik et al., 2020). Alternatively, such nutrients may be supplied by co-fermentation with conventional feedstocks although NGF substrate repression may have to be addressed, e.g., by using creA mutants in eukaryotes (Assis et al., 2021); cAMP-independent catabolite repressor (CcpA) in bacteria (Deutscher, 2008) or replacing the phosphotransferase system with the ATP-dependent sugar permease/kinase system (Zhang et al., 2015). The third option is to enhance the expression or activity of certain enzymes that are crucial for the biosynthesis of amino acids, sugars and nucleotides (Figure 9). In addition, for the assimilation of the highly oxidized feedstocks (CO_2_ and CO), energy source should be carefully chosen to make it industrially compatible, which can be light, chemical electron donors (e.g., H_2_, methanol, glucose), renewable electricity or their combinations.

Apart from medium compositions and reducing power, environmental factors such as medium pH and oxygen level are critical for microbial fermentation using NGFs. pH is particularly important for acetate and formate (Table 1). Increasing medium pH from 6 to 8–9 leads a drastic improvement in cell biomass growth and metabolite (lipids) production in *Y. lipolytica*. Importantly, this allows high acetate loading and yields much higher cell mass and lipid titres. Dry cell mass reached 37 g/L in small scale fed-batch fermentation when maintained at pH of 8 with 70 g/L initial acetic acid loading (Gao et al., 2020). Another important abiotic factor is oxygen. As we discussed previously, anaerobic fermentation has advantages for industrial bioproduction of certain types of products and carbon fixation. However, anaerobic conditions are incompatible with some production pathways or autotrophic systems that produce or require oxygen. Lastly, temperature control is critical for the fermentation of methanol and ethanol, which generates considerable heat and requires expensive cooling (Table 1). A solution to this problem is diverting reducing power towards anaplerotic metabolism (Schrader et al., 2009).

A one-fermenter-and-one-strain setup is the simplest but not always the best. System integration (e.g., multi-step bioprocess and microbial consortia) may drastically increase productivity and reducing production cost. Instead of directly using C1 gas to produce lipids, a two-step biosynthetic system may be more efficient than one-step system. A brilliant demonstration is the production of lipid by *Y. lipolytica* using dilute acetic acid (3%) derived from a gaseous biosynthetic system by acetogenic bacterium. A maximum lipid titre of 115 g/L was obtained, with substrate conversion rate of 0.16 g/g and productivity of 0.8 g/L/h (Xu J. et al., 2017). Co-cultures (Du et al., 2020) are a promising strategy which allows concurrent exploration of different genetic setups, thereby splitting pathways in two compartments. The anaerobic, non-photosynthetic mixotrophy was found to be a promising approach to increase product yield and decrease loss of CO_2_ (Claassens et al., 2016).

## Diversifying Value-Added Products in Biotechnology Industry

**FIGURE 10:**
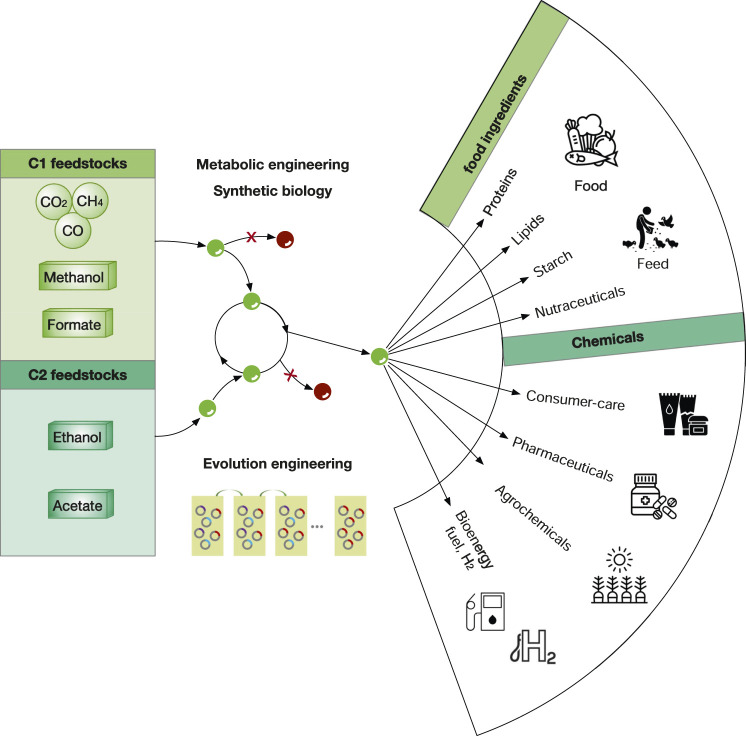
The overview of the bioeconomy using C1 and C2 feedstocks. Gaseous feedstocks (CO_2_, CO, H_2_) are in circles, liquid feedstocks (methanol, formate, ethanol and acetate) are in boxes. Green and red dots refer as intermediate metabolites and by-products. Metabolic engineering strategies include deleting the competing pathways to eliminate/minimize by-products. Elution engineering refers that the mutant strains with higher fitness (rings in red) will gradually dominate in the fermentation medium supplemented with unfavorable NGFs.

To date, microbial fermentation of NGFs have been widely explored to produce various valuable products including food ingredients (e.g., alternative proteins, lipids, starch, and nutraceuticals), specialty chemicals (e.g., flavors and fragrances), pharmaceuticals, agrochemicals (e.g., plant hormones) and bioenergy (fuels and H_2_) (Figure 10). Of note, many of these examples are still in laboratory stage and relative few examples are close to commercially viable cost. The key for commercialization is to reduce the manufacturing cost, in which high titres, production rates and yields (TRYs) are critical. In addition, the downstream purification should also be considered as it may contribute up to 15–70% of total manufacturing cost of microbial products (Stanbury et al., 2017). Nevertheless, as the technology in both metabolic engineering and bioprocess development is progressing very rapidly, we expect that more and more products derived from NGFs will reach industry in the next 5–10 years.

We have summarized some recent examples in Table 2. As there are too many studies in literature, here we choose only representative examples in various applications and far less than exhaustive. In addition, examples on biomass production (which can serve as food and feed) are not included in Table 2 as the yields are difficult to estimate. Currently, despite breakthrough in synthetic microorganisms, natural methylotrophs still dominate the current applications in using C1 gas (CO_2_, CO and CH_4_) and syngas. CO_2_ and H_2_ or syngas has been explored to produce single cell protein for food application (Molitor et al., 2019; Ruuskanen et al., 2021), small organic acids (acetate, lactate etc) (Liew et al., 2016) and alcohols (ethanol and butanol) using acetogens (Fackler et al., 2021).

**TABLE 2:** Bioproducts derived from C1 and C2 feedstocks using microbes and enzymatic reactions.

Feedstock	Bioproduct	Application	Microorganism/enzyme	Cultivation strategy	Titre (g/L)	Yield (g/g)^a^	References
CO_2_ and H_2_	Amylose	Food	Enzymes	Cell-free system	1.64	—^b^	Cai et al. (2021)
CO_2_ and pyruvate	Acetate	Bulk chemicals	Acetobacterium woodii DSM 1030	Continuous gas fermentation	59.2	—^b^	Kantzow et al. (2015)
CO_2_ and H_2_	Ethanol	Bulk Chemical	*Clostridium ljungdahlii*	Continuous gas fermentation	10 g/L/d	—	Gaddy et al. (2007)
CO_2_ and H_2_	Acetone	Bulk Chemical	*Clostridium autoethanogenum*	Continuous gas fermentation	3 g/L/d	—	Liew et al. (2022)
CO_2_ and H_2_	Isopropanol	Bulk Chemical	*C.autoethanogenum*	Continuous gas fermentation	3 g/L/d	—	Liew et al. (2022)
Syngas	n-Butanol	Fuel	*C. ljungdahlii*	Batch	0.148	—^b^	Köpke et al. (2010)
Methane	Methanol	Bulk chemicals	*Methylosinus trichosporium*	Fed-batch	1.34	—^b^	Hur et al. (2017)
Methane	Astaxanthin	Nutraceuticals	*Methylomonas* sp.	Batch	2.4 mg/g DCW	—^b^	Ye et al. (2007)
Methane	α-bisabolene	Consumer-care	*Methylotuvimicrobium alcaliphilum*	Batch	24.55	—^b^	Nguyen et al. (2021)
Methanol	L-glutamate	Food	*Bacillus methanolicus*	Fed-batch	60	—^b^	Heggeset et al. (2012)
Methanol	α-humulene	Consumer-care	*M. extorquens*	Fed-batch	1.65	0.03	Sonntag et al. (2015)
Methanol	Cadaverine	Precursor to polymers	*Bacillus methanolicus*	Fed-batch	11.3	—^b^	Nærdal et al. (2015)
Methanol	PHB	Biopolymers	*M. extorquens*	Fed-batch	52.9	0.12	Bourque et al. (1995)
Methanol	Pyruvate	Precursor to food and pharmaceuticals	*S. ceravisiae*	Batch	0.26	0.25	Dai et al. (2017)
Methanol and glycerol	Lovastatin	Pharmaceuticals	*P. pastoris*	Fed-batch	250.8	—^b^	Liu et al. (2018)
Acetate	Mevalonic acid	Precursor for pharmaceuticals/nutraceuticals	*E. coli*	Fed-batch	7.85	0.27	Xu et al. (2017b)
Acetate	β-Caryophyllene	Consumer-care	*E. coli*	Fed-batch	1.05	0.02	Yang and Nie, (2016)
Acetate	MNEI protein	Food	*E. coli*	Fed-batch	0.18	0.02	Leone et al. (2015)
Acetate	Lipids	Food or feed	*R. toruloides*	Batch	2.1	0.11	Huang et al. (2016)
Acetate	Lipids	Food or feed	*Y. lipolytica*	semicontinuous	115	0.16	Xu et al. (2017a)
Acetate	PHB	Biopolymers	*C. necator*	Fed-batch	43	—^b^	Garcia-Gonzalez and De Wever, (2018)
Acetate	PHB	Biopolymers	*E. coli*	Batch	1.27	0.25	Chen et al. (2018)
Acetate	Acetone	Bulk chemical	*E. coli*	Fed-batch	6.57	0.29	Yang et al. (2019)
Ethanol	PHB	Biopolymers	*E. coli*	Fed-batch	35.67	0.27	Sun et al. (2020)
Ethanol	PHB	Biopolymers	*E. coli*	Batch	1.1	0.11	Liang et al. (2021)
Ethanol and glucose	Amorphadiene	Drug precursor	*S. cerevisiae*	Fed-batch	40	—^b^	Westfall et al. (2012)

a Here, yield refers to mass of product per mass of substrate (g/g).

b Not determined or no data available.

Besides start-up companies like Air Protein Inc. (CA, United States), Solar Foods (Finland) and Deep Branch Biotechnology Ltd. (United Kingdom), there is one excellent pioneer in gas fermentation which is using anaerobic bacteria: LanzaTech Inc. (IL, United States), founded in 2005 in New Zealand. In May 2018, the world’s first commercial facility became operational which converts CO_2_ recycled from steel mill emissions to ethanol. This was made possible via a joint venture between LanzaTech and Shougang Group, a leading Chinese iron and steel producer. Located in Beijing, the plant currently has a capacity of 16 million gallons per year and more plants all over the world are being put into place and operation (Fackler et al., 2021). LanzaTech is also collaborating with companies like Unilever, Mibelle, L’Oréal, Lululemon, Zara and COTY to bring packaging, clothing, perfume, laundry detergent and household cleaners to the market—based on their carbon recycling technology that is branded as CarbonSmartTM. (https://www.lanzatech.com/#section-carbonsmart). In 2018, BASF Venture Capital announced to invest into LanzaTech to produce sustainable alcohols *via* industrial exhaust gas usage. Later in May 2021, both partners announced that they had reached the first milestone, namely successful conversion of CO and H_2_ to *n*-Octanol at lab-scale (https://www.basf.com/global/en/media/news-releases/2018/06/p-18-229.html).

In 2020, the partnership between Siemens Energy and Evonik has established the world’s first and fully automated CO electrolyzer which uses green power, CO_2_ and water to produce syngas. Subsequently, the syngas is used to produce butanol and hexanol with *Clostridium* strain in a 2,000 L bioreactor (Haas et al., 2018). It is projected that this so-called Rheticus project would produce 10,000 tons of butanol annually using 25,000 tons of CO_2_ as feedstock (https://www.siemens-energy.com/global/en/news/magazine/2020/rheticus-worlds-first-automated-co2-electrolyzer.html).

CO_2_ conversion to acetate is another highly attractive bioprocess that gains industrial interest. LanzaTech and IndianOil Ltd. (New Delhi, India) announced in 2020 that they are ready to take their process to a commercial scale. After the acetate production, acetate will be further upgraded using a heterotroph microalgae strain into lipids (high-value omega-3 fatty acids, e.g., docosahexaenoic acid or DHA, and fatty acids for biodiesel production) (https://iocl.com/NewsDetails/58912). Very recently, LanzaTech published the study that converts CO_2_ to acetone and isopropanol at a rate of ∼3 g/L/h at industrial pilot scale (Liew et al., 2022). Also, CO_2_ has been used to produce starch in the form of amylose and amylopectin in a cell-free system (Cai et al., 2021). However, the technology is currently in laboratory stage.

CH_4_ has been used to produce PHAs (Myung et al., 2017), methanol (Hur et al., 2017), single-cell proteins (Khoshnevisan et al., 2019), ectoine (Cantera et al., 2019), lipids (Fei et al., 2018), organic acids [e.g., lactic acid (Henard et al., 2016) and butyric acid (Garg et al., 2018)] and terpenoids [e.g., α-humulene and α-bisabolene (Nguyen et al., 2021)]. More examples can be found in the recent article (Gęsicka et al., 2021). In 2020, Calysta, a Californian start-up company, and Adisseo, a worldwide animal nutrition leader, announced a joint venture named Calysseo (https://calysseo.com/) to develop their technology to commercial scale in China. It aims to operate in 2022 to deliver 20,000 tons of methane-based animal feed protein annually. Another promising industrial player in this field is Unibio (https://www.unibio.dk). Its methanotrophic biomass products are protein rich and the amino acid profile is favorable for the feed of fish and livestock such as pigs and chicken.

Formate has potentials in producing fuels, value-added chemicals, and microbial proteins (Yishai et al., 2016) but has not been explored to date due to high cost. C2 feedstocks especially acetate have been used to produce many value-added chemicals (derived from acetyl-CoA) as we covered previously. Here, we further selected some recent examples and summarized in Table 2.

## Conclusion

The COP26 summit has reiterated the urgency to reduce carbon emission. Microbial utilization of NGF offers a nice solution to alleviate this issue by circulating CO_2_ in a closed loop and generating value-added products. Feasibility in this strategy has been validated by both academy and industry. As the technological challenges in microbial strain engineering and bioprocesses are being addressed and overcome gradually, we expect that biorefinery of NGF will contribute to economy and, more importantly, sustainability. We also welcome more students, scientists, engineers and policy-makers to join the global efforts to develop new bioprocesses for a better and more sustainable future.
